# 3D-printed microfluidics integrated with optical nanostructured porous aptasensors for protein detection

**DOI:** 10.1007/s00604-021-04725-0

**Published:** 2021-02-04

**Authors:** Sofia Arshavsky-Graham, Anton Enders, Shanny Ackerman, Janina Bahnemann, Ester Segal

**Affiliations:** 1 Department of Biotechnology and Food Engineering, Technion – Israel Institute of Technology, Haifa, Israel; 2 Institute of Technical Chemistry, Leibniz University Hannover, Hanover, Germany; 3 The Russell Berrie Nanotechnology Institute, Technion – Israel Institute of Technology, Haifa, Israel

**Keywords:** Porous silicon, 3D-printing, Polyacrylate, PDMS, Microfluidics, Biosensor

## Abstract

Microfluidic integration of biosensors enables improved biosensing performance and sophisticated lab-on-a-chip platform design for numerous applications. While soft lithography and polydimethylsiloxane (PDMS)-based microfluidics are still considered the gold standard, 3D-printing has emerged as a promising fabrication alternative for microfluidic systems. Herein, a 3D-printed polyacrylate-based microfluidic platform is integrated for the first time with a label-free porous silicon (PSi)–based optical aptasensor via a facile bonding method. The latter utilizes a UV-curable adhesive as an intermediate layer, while preserving the delicate nanostructure of the porous regions within the microchannels. As a proof-of-concept, a generic model aptasensor for label-free detection of his-tagged proteins is constructed, characterized, and compared to non-microfluidic and PDMS-based microfluidic setups. Detection of the target protein is carried out by real-time monitoring reflectivity changes of the PSi, induced by the target binding to the immobilized aptamers within the porous nanostructure. The microfluidic integrated aptasensor has been successfully used for detection of a model target protein, in the range 0.25 to 18 μM, with a good selectivity and an improved limit of detection, when compared to a non-microfluidic biosensing platform (0.04 μM vs. 2.7 μM, respectively). Furthermore, a superior performance of the 3D-printed microfluidic aptasensor is obtained, compared to a conventional PDMS-based microfluidic platform with similar dimensions.

**Supplementary Information:**

The online version contains supplementary material available at 10.1007/s00604-021-04725-0.

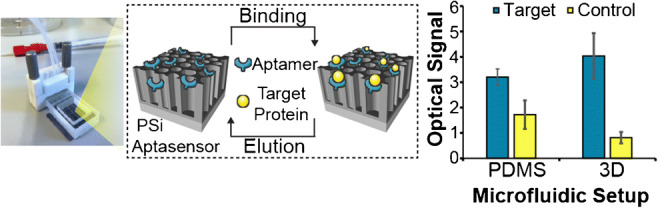

## Introduction

Microfluidic systems and their integration with biosensors are extensively studied for construction of lab-on-a-chip platforms [1, 2]. The miniaturization of such systems reduces sample and reagent volume, shortens the analysis time, and enables high-throughput detection, portability, and reduced costs [1, 2]. Importantly, microfluidics improves the mass transfer to the biosensor surface, resulting in a higher sensitivity compared to traditional biosensing setups [3–5]. Nowadays, polydimethylsiloxane (PDMS) is the most used polymer for microfluidics fabrication. It is commonly constructed by casting on a master template featuring the microfluidic design, which is fabricated by soft lithography techniques. Thus, it requires sophisticated instrumentation and high costs, while the translation to commercial scale is challenging [6, 7]. Importantly, a change in the microfluidic design cannot be performed without repeating the whole fabrication process, which poses a significant barrier for rapid prototyping [6, 7]. The technological advancement of 3D-printing, which facilitates rapid and fully digital prototyping of complex 3D microstructures in a one-step process, has positioned it as a promising alternative to traditional manufacturing methods [8]. 3D printing also lowers the costs and the manufacturing time compared to soft lithography and enables the fabrication of multiple devices at the same time [6, 8]. This facilitates a flexible investigation of microfluidic device designs for any desired application [6, 9].

For integrating 3D-printed microfluidics with biosensors, one must consider the resolution of the printer (often on the range of tens of microns), the resulting surface roughness of the printed device, its deformation, and resistance to harsh conditions [8]. Thus, bonding to a biosensor surface can be achieved by indirect methods, via an intermediate layer, which can be performed at comparatively mild conditions at a lower surface quality [10, 11]. As an intermediate layer, UV-curable adhesives are most commonly used, since they require only a short UV irradiation for curing and can be operated at ambient conditions [11]. The adhesive can be applied to the substrates by means of capillary forces [12, 13], scaffold micropillars [14], or by stamping technique [15–18]. In the stamping technique, a thin layer of the adhesive is first spun on a flat wafer. Then, the microfluidic part is stamped on the adhesive-coated wafer, resulting in adhesive transfer to the microfluidic part, which can be then bonded to a second sealing part [15, 16]. This method allows for the creation of a thin (<3 μm) adhesive intermediate layer that does not interfere with the microchannel area and was successfully applied for relatively smooth and even surfaces (such as glass, silicon, and SU-8) [10, 15, 18]. Yet, for biosensing applications, this method was only demonstrated with microfluidic glass chips used for surface plasmon resonance [16].

To date, there are only a handful of reports that combine 3D-printed microfluidics with aptasensors [19–21], and herein, we present for the first time the integration of a 3D-printed microfluidic device with a generic label-free optical porous silicon (PSi) aptasensor. The nanostructured PSi scaffold is used as the optical transducer, and binding of the target analyte to surface-immobilized aptamers, used as capture probes, is detected in real time by monitoring reflectivity changes of the PSi [22–27]. To date, PSi-based optical biosensors have been only integrated with PDMS-based microfluidics, fabricated by soft lithography [28–34]. In the present work, we develop a straightforward and low-temperature bonding method for the integration of the PSi-based aptasensor with 3D-printed polyacrylate-based microfluidics, utilizing a UV-curable adhesive as an intermediate layer. The method is derived from the stamping technique and creates a thin adhesive layer. It should be emphasized that in contrast to previous works, in the present work we are integrating a relatively rough surface of a 3D-printed polyacrylate microchannels with a delicate highly porous nanostructure. As a proof-of-concept to demonstrate the biosensing capabilities of the platform, we use a model aptasensor: oxidized PSi Fabry-Pérot thin film is functionalized with an anti-his tag aptamer, and a 60 kDa his-tagged protein is used as a target. The aptasensor sensitivity is evaluated and the selectivity is characterized by exposure to several non-target proteins as well as to bacteria lysate samples. Importantly, we compare the aptasensor performance in the 3D-printed device to that of PDMS microchannels with similar dimensions, as well as to non-microfluidic setups.

## Experimental

### Materials

Heavily doped p-type Si wafers (<100>-oriented, ~0.95 mΩ∙cm resistivity) were purchased from Sil’tronix Silicon Technologies (Archamps, France). Ethanol absolute was supplied by Bio-Lab ltd (Jerusalem, Israel). Aqueous HF (48%), (3-aminopropyl)triethoxysilane (APTES), succinic acid, dimethylsulfoxide (DMSO), N-(3-dimethylaminopropyl)-N′-ethylcarbodiimide hydrochloride (EDC), Tris base, and all buffer salts were purchased from Merck (Darmstadt, Germany). All solutions were prepared with Milli-Q water (ddH_2_O, 18.2 MΩ∙cm). Polydimethylsiloxane (PDMS) was prepared from Sylgard® 184 Silicon Elastomer kit, purchased from Dow Corning (Midland, USA). Anti-his tag aptamer 6H7 (5′-GCT ATG GGT GGT CTG GTT GGG ATT GGC CCC GGG AGC TGG C-3′) [35] was purchased with a 5′-amino modification from Integrated DNA Technologies (Coralville, USA). Recombinant his-tagged protein, domain 2 of extracellular endo-alpha-(1->5)-L-arabinanase 1 (from *Geobacillus stearothermophilus T-6*) (D2), and a non-target version without a his tag (named D2N) were both produced and purified using *Escherichia coli* BL21 cells (the process is detailed in the Supplementary Information). Trypsin from porcine pancreas was obtained from Merck, and *E. coli* K12 was generously supplied by Prof. Sima Yaron, Technion. Luria-Bertani (LB) medium was prepared by dissolving 5 g of NaCl, 5 g of yeast extract, and 10 g of tryptone in 1 L of ddH_2_O. *E. coli* lysate suspensions were prepared as previously described [36] (see Supplementary information for more details). Selection buffer (SB) was composed of 50 mM K_2_HPO_4_, and 150 mM NaCl (pH 7.4) and elution buffer was composed of 50 mM K_2_HPO_4_, 150 mM NaCl, and 1 M imidazole (pH 7.4).

### Fabrication of oxidized PSi nanostructures

PSi Fabry-Pérot thin films were fabricated from a highly doped p-type crystalline Si wafers, using a two-step anodic electrochemical etching process, as previously described [37]. The electrochemical etching was performed at a constant current density of 375 mA cm^−2^ for 30 s in a 3:1 (v/v) solution of aqueous HF (48%) and ethanol, respectively, followed by thermal oxidation at 800 °C for 1 h in ambient air. For further details, please see Supplementary information and Table S1.

### Design and fabrication of 3D-printed microfluidic devices

**Fig. 1:**
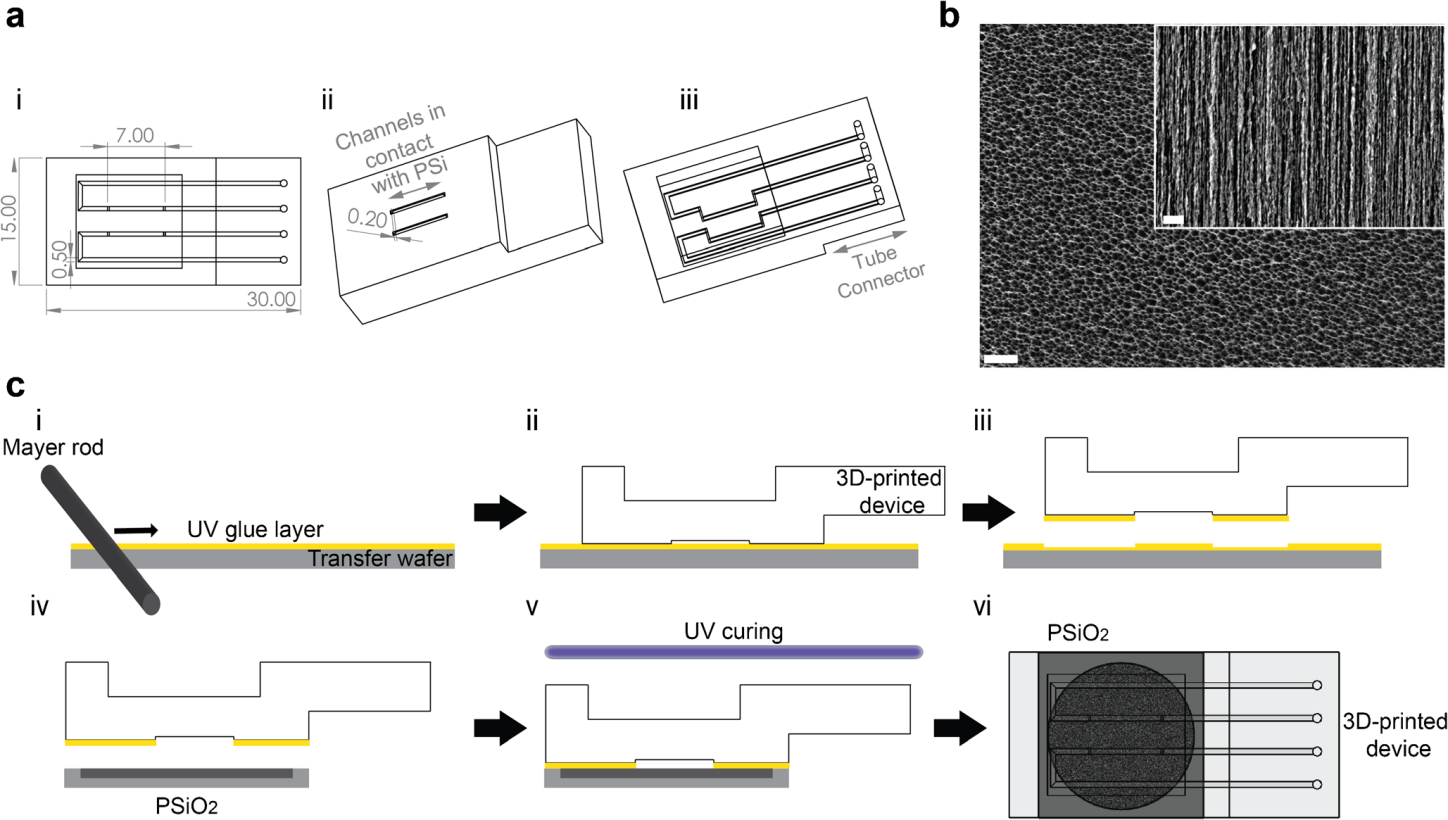
The microfluidic design, the PSi nanostructure, and the bonding method. **a** The 3D-printed microfluidic device design presented in (i) top; (ii) bottom-side, and (iii) top-side views. Dimensions are in mm units. **b** Top-view and cross section HRSEM micrographs of an oxidized PSi nanostructure (the inset presents a cross section view; scale bars are 400 nm). **c** The bonding method of the 3D-printed microfluidic device and the PSi: (i) a UV-curable adhesive is spread on a transfer wafer with a Mayer rod; (ii) the 3D-printed device is placed on top; (iii) the glue transfers to the device; (iv) the microfluidic device is carefully placed on top of the PSi chip; (v) the combined device is UV cured for 30 min; and (vi) the resulting integrated device

The microfluidic device was designed in SolidWorks software (Dassault Systèmes) and contained two separate microchannels, with dimensions of 200 μm in height and 500 μm in width, spaced 2.5 mm apart. The microchannel length to be in contact with the PSi was 7 mm, while the rest passed within the device. Above the contact area, a measurement window was created, reducing the thickness of the device by 2.8 mm (see Fig. 1). The designed devices were printed using 3D Systems Projet MJP 2500 Plus multijet printer. Polyacrylate-based photopolymer material (VisiJet M2R-CL, 3D Systems, Rock Hill, USA) and hydroxylated wax (VisiJet M2 Sup, 3D Systems, Rock Hill, USA) were used as the printing and support materials, respectively. The microchannels were located at the bottom layer of the printing plane and in parallel to the printing direction. The printer resolution was 32 μm and a deviation of 10% in size was reported for features with sizes of 100 to 200 μm [38]. After printing, the devices were subjected to several postprocessing steps, as previously described (see Supplementary information for more details) [6, 39].

### Integration of PSi films with 3D-printed polyacrylate microfluidic devices

Prior to bonding the 3D-printed device to the PSi film, the devices were first gently polished with a standard grid paper (1000 grit), washed with water and soap, and flattened at 60 °C by applying a pressure of ~38 kPa for 1 h. During this step, the measurement window was filled with a fitting square that ensures the alignment of the microchannel bonding area to the PSi. Next, UV-curable adhesive (Norland Optical Adhesive 72, Norland Products Inc., Cranbury, USA) (50 μL) was spread on a transfer wafer with a K hand coater no. 2 (Printcoat Instruments, Litlington, UK) and the printed device was placed on top of the adhesive layer two times for the glue transfer. Finally, the device was carefully placed on top of carboxylated PSi films, followed by UV curing at 365 nm (1.5 mW cm^−2^) for 30 min (VL-6.LC UV lamp 365/254 nm 6 W, Vilber Lourmat, Collégien, France).

### Integration of PSi films with PDMS microchannels

PDMS microchannels were fabricated based on a 3D-printed polyacrylate-based template, with 200-μm-high and 500-μm-wide microchannels. PDMS was prepared by mixing the polymer and the cross-linker at a 10:1 ratio, respectively, followed by curing at 60 °C overnight. Then, the inner surface of the PDMS microchannels was treated with corona for 40 s, using a laboratory corona treater (BD-20 V Electro-Technic Products, Chicago, USA). The activated PDMS was then placed on top of carboxylated PSi films, followed by baking at 90 °C for 4 h [28, 29].

### Characterization of integrated devices

The 3D-printed microfluidic devices, integrated with the PSi, were characterized by several methods. Standard food color solutions (E124 and E133) were introduced into the microchannels to visualize possible leakage. It should be noted that these dyes could be successfully removed by flushing the microchannels with water and no microchannel staining was apparent. The integrated devices were imaged with an upright optical microscope Olympus BX51 (Olympus). Cross sections of the integrated devices were characterized by Carl Zeiss Ultra Plus high-resolution scanning electron microscope (HRSEM), at an accelerating voltage of 1 keV. The cross-sectioned samples were prepared by embedding the devices in epoxy (EpoFix resin, Struers, Cleveland, USA), which was refilled several times under vacuum (1 Torr) to remove any air, followed by curing at room temperature for 48 h. The cured epoxy block was sectioned using an IsoMet™ low speed saw (Buehler, IL, USA) and polished in EcoMet™ 3 variable speed grinder-polisher (Buehler, IL, USA) with sandpaper with increasing grit, as well as an Alumina Suspension (50 nm Neutral, Akasel, Denmark). Finally, the cross-sectioned samples were sputtered with carbon.

### Functionalization of oxidized PSi films

Amino-terminated anti-his tag aptamers, 6H7, were grafted onto the oxidized PSi films using carbodiimide coupling chemistry [36]. Briefly, the oxidized PSi films were incubated for 1 h in APTES solution (in toluene, 42 mM), followed by a thorough rinsing with toluene, ethanol, and acetone. Next, an annealing step was performed at 100 °C for 15 min. The PSi film was cooled down to room temperature and subsequently incubated in a solution of succinic acid (0.17 M) and NaHCO_3_ (6 mM) in DMSO for 30 min, followed by washing with DMSO and ddH_2_O and drying it under a stream of nitrogen. After this step, the carboxylated PSi was integrated in the microfluidic device, and the remaining functionalization steps were carried out inside the microchannels.

The microchannels were first washed with EtOH (50%v/v) in ddH_2_O for 5 min to remove any air bubbles inside the channels, followed by subsequent washing with SB buffer at 100 μL min^−1^ for 10 min. Next, EDC in SB buffer (10 mg mL^−1^) was introduced at 30 μL min^−1^ for 30 min, followed by introduction of aptamer (75 μM, 250 μL) at 30 μL min^−1^. The aptamer was then allowed to react with the surface for 1 h without flow. Subsequently, the microchannel was washed with Tris (50 mM, pH 7.4) at 30 μL min^−1^ for 15 min to deactivate any remaining reactive EDC groups on the surface.

### Biosensing experiments

For biosensing experiments, the microfluidic integrated PSi was fixed on a motorized linear translation stage (Thorlabs, Inc., NJ, USA) and four different spots, all spaced out at 1.5 mm apart, were monitored on each microchannel in every experiment. A syringe pump (Fusion 200, Chemyx, TX, USA) was used to control the flow rate. The 3D-printed microfluidic device was connected to tubes through a Dolomite 4-way microfluidic connector and a 4 mm top interface (The Dolomite Center Ltd., Royston, UK). Female-to-male Luer Assy and flangeless fittings (IDEX Health and Science LLC, Middleboro, USA) were used to connect the tubes to a syringe. For the cell setup, the PSi aptasensor was fixed in a custom-made plexiglass cell, using an O-ring.

RIFTS method was used to monitor in real time the reflectivity changes of the PSi-based aptasensor [25, 36]; the latter presenting a Fabry-Pérot interference fringe pattern attained from light reflecting from the top and bottom interfaces of the PSi. The fringe maxima are described by the Fabry-Pérot relationship:

$$ m\lambda =2 nL $$

where *m* is the spectral order, *λ* is the wavelength of the incident light, *n* is the average refractive index (RI) of the porous layer, and *L* is the thickness of the porous layer. The term 2*nL* is referred to as the effective optical thickness (EOT) and is only a function of the average RI of the porous layer, as the thickness is constant. A schematic illustration of the method is presented in Figure S1.

Interferometric reflectance spectra were collected with a charge-coupled device (CCD) spectrometer (Ocean Optics, USB 4000) fitted with an objective lens and coupled to a bifurcated fiber-optic cable. A tungsten light source was focused on the microchannel or sample with a spot size of approximately 1 mm^2^, perpendicular to the surface. The experimental setup is presented in Figure S2 (Supplementary information). The spectral acquisition and the stage movement were controlled with a LabView software (National Instruments). The spectra were acquired at an integration time of 30 ms and with a scan average of 160, every ~1.1 min. Fast Fourier transformation (FFT) was performed at a wavelength range of 450–900 nm, as previously described by Massad-Ivanir et al [40]. This results in a single peak, wherein position along the x-axis equals the EOT of the porous layer and is linearly correlated to the RI changes of the PSi. For the flow experiments, the microchannel was washed with elution buffer for 30 min, followed by a 1 h wash with SB buffer, to allow the aptamer to properly fold to its active 3D structure. Next, the protein samples (0.25 to 18 μM, in SB buffer) or lysate (with a protein content of 1 mg mL^−1^) was introduced for 1 h, after which the surface was washed with SB for 30 min. To regenerate the surface for additional experiment, elution buffer was introduced for 30 min, followed by SB wash for 30 min. Each microchannel was used for two biosensing cycles. Flow rate was kept at 30 μL min^−1^ for all steps. For the static experiment in the cell setup, the buffers (~5 mL) were introduced to the cell with a syringe and allowed to incubate for the same amount of time. Protein sample (100 μL of 1 μM in SB) was injected to the cell with a pipette and allowed to incubate for 1 h. The buffer washing steps were performed with 10 mL of the buffers.

The data is presented as relative ΔEOT, defined as

$$ \frac{\Delta  {EOT}_t}{EOT_0}=\frac{EOT_t-{EOT}_0}{EOT_0} $$

where *EOT*_0_ is the average EOT signal at the baseline acquisition with SB buffer prior to protein introduction, and *EOT*_*t*_ is the average EOT signal at the last 5 min of protein incubation/flow phase. Calibration curve was fitted with a sigmoidal curve, according to the equation:

$$ R={R}_{max}\frac{\left[C\right]}{\left[C\right]+{K}_D} $$

where *R* is the relative EOT signal, [*C*] is the target concentration, *R*_max_ is the maximal response signal attained for [*C*] → ∞, and *K*_D_ is the apparent dissociation constant. *R*_max_ and *K*_D_ are equal to (7.7 ± 0.3) × 10^−3^ (as ΔEOT/EOT_0_) and 0.9 ± 0.1 μM, respectively.

The signal-to-noise ratio (SNR) values are calculated as the ratio of the relative EOT signal to the standard deviation (σ) of the baseline in SB prior to protein introduction. The latter equals to 0.08 × 10^−3^, 0.07 × 10^−3^, and 0.09 × 10^−3^ (as ΔEOT/EOT_0_) for the cell setup, PDMS, and 3D-printed microfluidic setups for 1-μM protein experiments, respectively. Limit of detection (LoD), the lowest target concentration which can be reliably distinguished from the background noise, is calculated by extrapolation from the fitted curve of the concentration when the optical signal is equal to 3∙σ. For 3D-printed microfluidic setup, the average σ for all experiments is equal to 0.11 × 10^−3^ (as ΔEOT/EOT_0_). Limit of quantification (LoQ) is calculated as the concentration which yields an optical signal equal to 10∙σ. Relative standard deviation (%RSD) is calculated for each experimental set as the standard deviation divided by the averaged relative EOT change.

### Statistical analysis

In the microfluidic setups, repeats were performed on at least two independent microchannels, in which four different spots were measured. For the cell setup, repeats were performed on at least three different aptasensors. All data is presented as the mean of *n* ≥ 3 with standard deviation of the mean. For statistical evaluation, unpaired *t* test was performed with two-tailed distribution and unequal variance. *p* values below 0.05 were considered for significant difference between two groups.

## Results and discussion

### Microfluidic design and integration with PSi films

The 3D-printed polyacrylate microfluidic device design is presented in Fig. 1a. Each device contains two separate microchannels, spaced out at 2.5 mm apart, with a width of 500 μm and height of 200 μm. Out of the total microchannel length (55.6 mm), only 7 mm are in contact with the PSi to minimize the required bonding area with the highly porous surface, while allowing for multi-spot optical measurements along the channel. A measurement window is created above the contact area to reduce the thickness of the polyacrylate material in the optical measurement area.

The preferable direct bonding of the microfluidic device to the PSi is not feasible owing the combination of the rough surface of the 3D-printed polyacrylate and the delicate porous nanostructure. The latter is characterized by a high porosity of ~75% and cylindrical pores with a diameter of ~50 nm, as depicted in the high-resolution scanning electron microscopy (HRSEM) micrographs in Fig. 1b (and summarized in Table S1, Supplementary information). Thus, we use an intermediate-layer bonding approach, derived from the stamping technique [15], utilizing a UV-curable adhesive as the intermediate. The latter is used as these adhesives only require a short UV irradiation for curing and as such avoid harsh conditions, which may damage the fragile silicon scaffold. Prior to the bonding process, the 3D-printed device, which often suffers from bending and a roughened surface due to the printing resolution [6, 39], is subjected to several alignment steps for its flattening. These include gentle gridding and flattening by applying a pressure of ~38 kPa at 60 °C for 1 h.

**Fig. 2:**
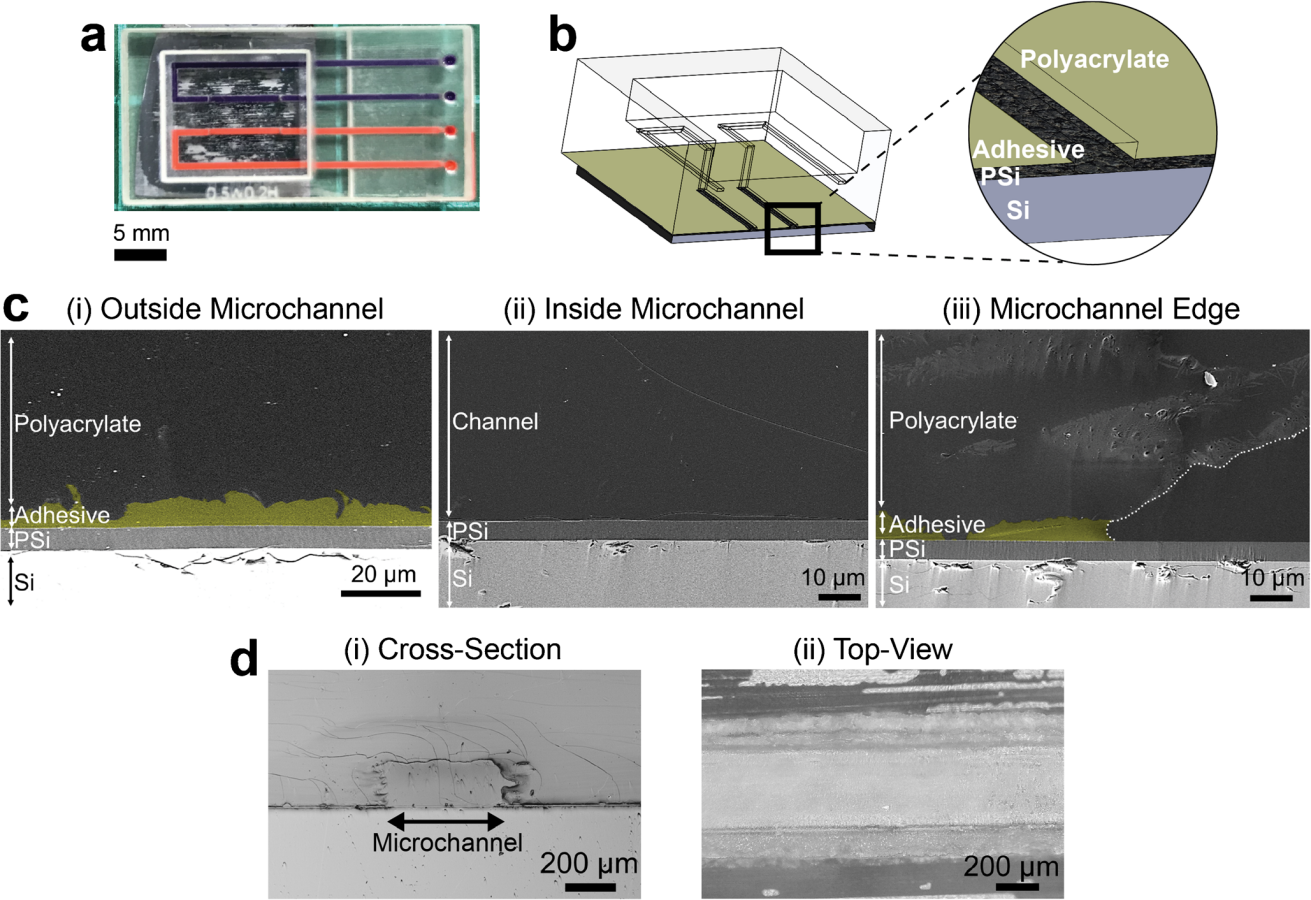
Characterization of the 3D-printed microfluidic integrated PSi device: **a** Leakage study by dye flow in the microchannels; **b** illustration of the integrated device cross section, showing its different layers; **c** HRSEM images of a cross-sectioned device, presenting an area (i) outside the microchannel, (ii) inside the microchannel, and (iii) the microchannel edge, which is marked in white dashed line. No adhesive is found inside the microchannel, whereas a continuous 5 ± 2-μm-thick adhesive layer can be observed outside of the microchannel, between the PSi and the polyacrylate device. Note: for clarity, the adhesive layer is false-colored in yellow. **d** (i) Cross section and (ii) top-view optical micrographs of the integrated device, demonstrating the roughness of the polyacrylate material at the microchannel edges

Figure 1c presents the multi-step integration process; first, the UV-curable adhesive is spread on a transfer wafer with a Mayer rod (Fig. 1c-i), which creates a thin and uniform adhesive layer with a thickness of 12 μm. In the next step, the microfluidic device is placed on top of the coated transfer wafer (Fig. 1c-ii) and the glue is observed to spread throughout the device (Fig. 1c-iii). This step is repeated twice, and then the microfluidic device is carefully placed on top of the PSi chip (Fig. 1c-iv), followed by UV curing for 30 min (Fig. 1c-v). It should be noted that the UV curing duration was not optimized and can be potentially shortened. No leakage or microchannel clogging is observed upon introduction of a dyed solution into the microchannels (Fig. 2a), confirming successful bonding of the substrates. The effect of the bonding method on the microchannels and the porous regions is characterized by HRSEM imaging of the device cross section, illustrated in Fig. 2b. Outside of the microchannel, a continuous 5 ± 2-μm-thick adhesive layer is observed between the porous layer and the top polyacrylate device (see Fig. 2c-i). The microchannel is completely free from adhesive, even in the interface regions of the channel’s edges (Fig. 2c-ii and iii, respectively). Notably, the integrity of the bonding between the layers is maintained through the harsh cross-sectioning procedure for the HRSEM, as well as >1 year postbonding (see Figure S3, Supplementary information). These demonstrate the bonding strength and suggest that the bonding method does not limit the long-term stability of the integrated devices. Thus, the latter is dictated by the aptasensor characteristics, i.e., capture probe and its immobilization chemistry. The relatively thick adhesive layer [10, 15, 16] is required to ensure a good contact between the porous substrate and the 3D-printed device. The layer printing technique and the 32-μm resolution of the printer result in a rough polyacrylate surface, as can be seen in Fig. 2d. Yet, this adhesive layer thickness is negligible compared to the current channel height and is compatible with the lowest microchannel dimensions allowed by the printer (64 μm, data not shown). The presented bonding method is straightforward compared to other reported techniques [10], and can be readily performed and adapted to other device configurations.

### Biosensing experiments and performance

In order to study the biosensing performance of the integrated platform, we use a model aptasensor, which we thoroughly characterized in our previous work, to allow a proper comparison of the biosensing results [36, 37]. An anti-his tag aptamer, 6H7 [36, 37], is immobilized onto the PSi film by the standard amino-silanization and carbodiimide coupling chemistry, as we previously described [36, 37]. The amino-silanization and carboxylation steps are performed prior to microfluidic device bonding, whereas the subsequent immobilization stages should be executed inside the microchannel. The UV curing during the bonding process is found to affect the functionality of the aptamers as capture probes (see Figure S4, Supplementary information), possibly due to modification of DNA bases [41].

**Fig. 3:**
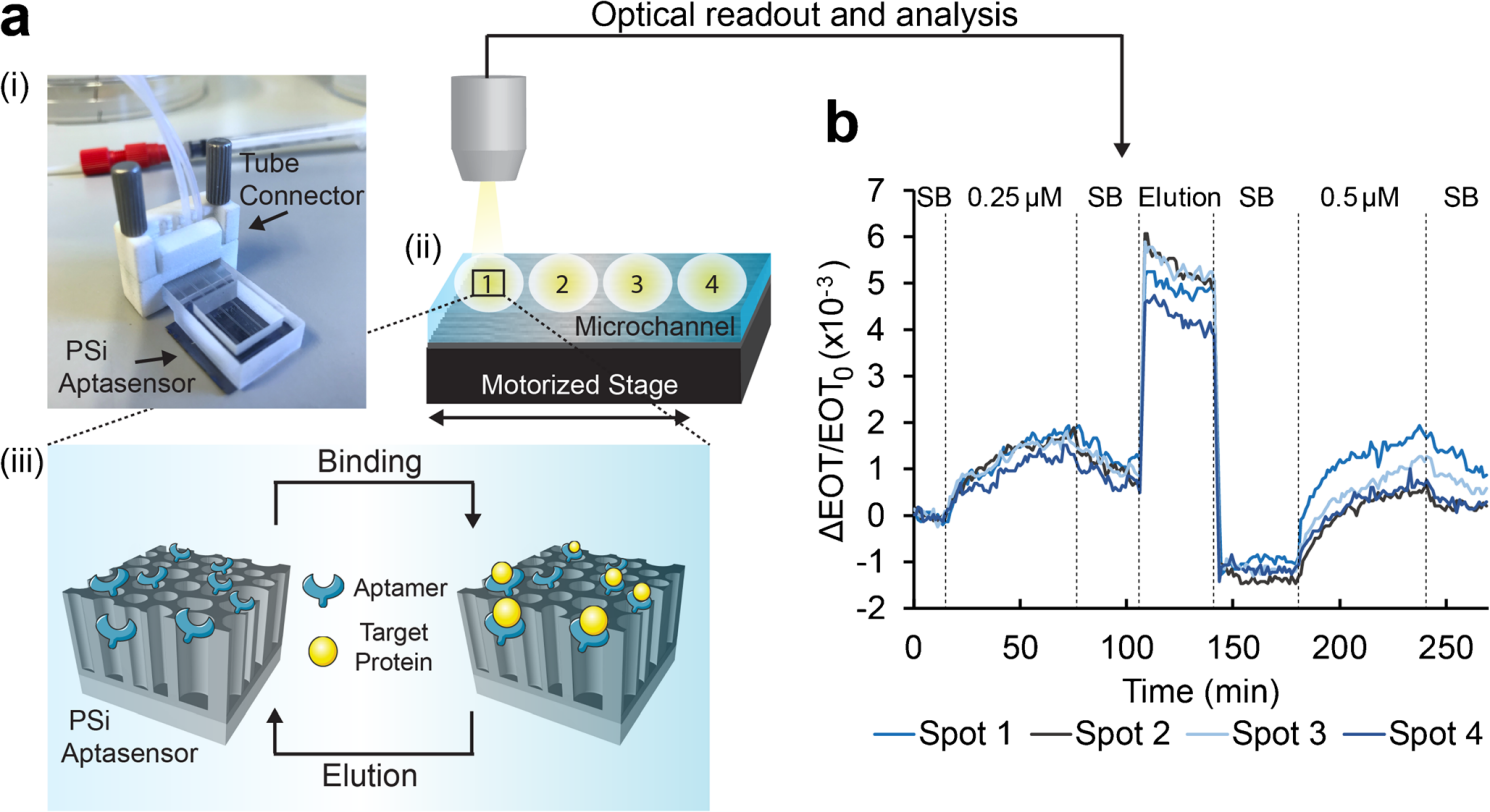
Biosensing experiments using the 3D-printed microfluidic integrated PSi aptasensor. **a** (i) The microfluidic device is connected to tubes with a designated tube connector; (ii) it is fixed on a motorized stage, enabling optical monitoring of four different spots along a single microchannel. (iii) The anti-his tag PSi-based aptasensor is used to detect the target D2 protein and can be easily regenerated for several subsequent uses by exposure to elution buffer, containing imidazole. **b** Relative EOT changes vs. time upon introduction of D2 protein solutions. First, a baseline is acquired in selection buffer (SB), followed by introduction of 0.25 μM protein at a flow rate of 30 μL min^−1^ for 1 h, and wash with SB. Subsequently, the biosensor is washed with an elution buffer, resulting in the aptasensor regeneration for a subsequent experimental cycle, using a protein concentration of 0.5 μM

For the biosensing experiments, a 60 kDa his-tagged protein from the Arabinanase family (termed as D2) is used as the target. The protein solution is introduced into the microchannels using a custom-designed 3D-printed tube connector and the reflectivity from the PSi is collected throughout the experiment from four different spots along the channel, as presented in Fig. 3a (see Figure S2, Supplementary information, for a complete description of the experimental setup). Figure 3b displays the results of two consecutive biosensing cycles with increasing protein concentrations, performed on the same aptasensor, where changes in the relative EOT values are plotted vs. time. Initially, the aptamer’s selection buffer (SB) is introduced into the microchannel to allow proper folding of the aptamers and to acquire the initial EOT baseline. Upon the introduction of the D2 protein (0.25 μM), the relative EOT signal increases to a value of (1.6 ± 0.2)×10^−3^ (as ΔEOT/EOT_0_) (equivalent to a net EOT change of 27 ± 2 nm), corresponding to the protein infiltration into the porous layer and binding to the immobilized aptamers. The signals collected from the different spots along the microchannel present a similar behavior, with a deviation of <9%. This suggests that a uniform bonding of the 3D-printed microfluidic device to the PSi is achieved, whereas the observed deviation is attributed to the variation of the PSi nanostructure along the microchannel, resulting from the anodization reaction, as well as the manual adjustment of the reflectivity measurement position from each spot. Nevertheless, no correlation is found between the sequential location of the spot along the microchannel and the optical signal value.

To release the bound protein, the aptasensor surface is washed with an elution buffer containing imidazole; the latter serves as a competitive agent, replacing the his-tagged proteins bound to the tethered aptamers [42]. Indeed, the EOT signal is observed to rapidly decrease, indicating the release of the bound proteins. Yet, it should be noticed that the signal decreases below the initial baseline possibly due to conformational changes of the immobilized aptamers. Aptamers 3D folding greatly depends upon their environment and as such saturating the biosensor with imidazole molecules, replacing the large proteins, may lead to prominent changes in the aptamer 3D structure, as we previously encountered [29]. The aptasensor is then successfully reused for an additional biosensing cycle of the D2 protein at a higher concentration (0.5 μM), and a greater relative EOT increase of (2.3 ± 0.4)×10^−3^ (as ΔEOT/EOT_0_) (corresponding to a net EOT change of 37 ± 7 nm) is observed.

**Fig. 4:**
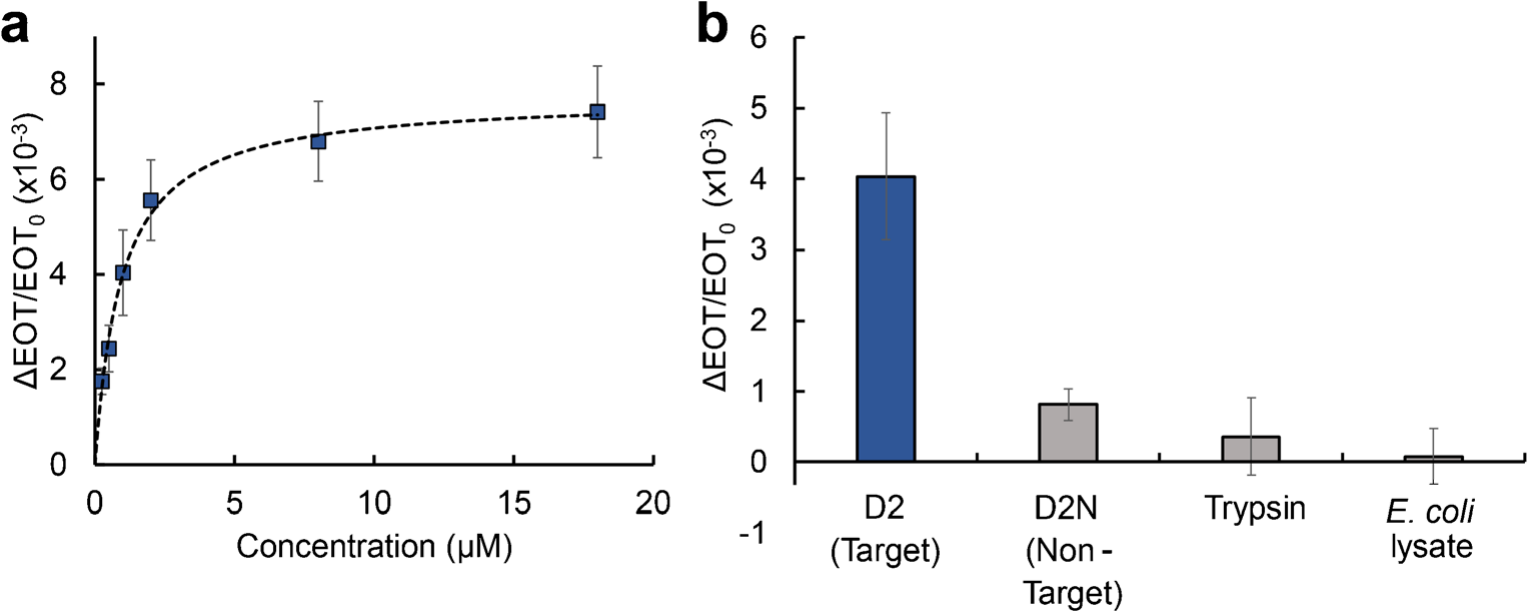
Averaged relative EOT changes upon exposure of the 3D-printed microfluidic integrated aptasensor to **a** different concentrations of the target D2 protein; **b** D2 and non-target proteins. Protein concentrations are 1 μM for D2 and D2N (D2 without a his tag), 9 μM for trypsin, and 1 mg mL^−1^ protein content in the *E. coli* lysates

Figure 4a presents the averaged relative EOT changes for the target D2 protein at a concentration range of 0.25 to 18 μM. The lowest measured concentration is 0.25 μM with a relative EOT increase of (1.8 ± 0.3) ×10^−3^ (as ΔEOT/EOT_0_) with a signal-to-noise ratio (SNR) of 19 ± 7. The curve is fitted with a sigmoidal curve (*R*^2^ = 0.915), and accordingly, the *K*_D_, the apparent dissociation constant, is estimated as 0.9 ± 0.1 μM and is on the same order of magnitude as previous reports (*K*_D_ of 4.6 μM [36]).

Table 1 summarizes the analytical performance of the integrated aptasensor and provides a comparison to a non-microfluidic system (in which the same aptasensor is used for detection of a different his-tag protein [37]). Notably, a significant improvement of ~70-fold in the limit of detection is achieved in the 3D-printed microfluidic system. This is mainly attributed to the microfluidic integration and the flow configuration during the biosensing experiment, as discussed in the next section. The different target may also influence the performance, with a better accessibility of the histidine sequence in the protein structure for the binding aptamer.

**Table 1:** Analytical results of the 3D-printed microfluidic integrated aptasensor, compared to a similar aptasensor in a non-microfluidic setup (cell). Assay time is similar in both systems to allow proper comparison

	3D-printed microfluidic setup	Non-microfluidic (cell) setup
Detection range (μM)	0.25–18	5–56
SNR*	19 ± 7	16 ± 6
LOD (μM)	0.04	2.7
LOQ (μM)	0.16	5.5
%RSD	12–22	6–27

*For lowest measured target concentration

Figure 4b compares the averaged relative EOT signal for the target D2 protein to non-target proteins. While 1 μM of the target protein induces a relative EOT change of (4.0 ± 0.9)×10^−3^ with SNR value of 41 ± 17, exposure of the biosensor to a similar protein with no his-tag group (D2N) results in a significantly lower signal of only (0.8 ± 0.2)×10^−3^, with a lower SNR value of 13 ± 6. This demonstrates that the optical signal obtained for the target D2 is ascribed mainly to specific binding of the his-tag sequence of the protein to the tethered aptamers. Exposure of the aptasensor to a higher concentration of trypsin, and *E. coli* lysates (a complex protein mixture, which simulates best a control for his-tagged protein purification applications) with a substantial non-target protein content of 1 mg mL^−1^, induces even lower signal changes [(0.4 ± 0.6)×10^−3^ and (0.1 ± 0.4)×10^−3^, respectively]. These results demonstrate that the selectivity of the aptasensor is not compromised by the integration with the printed microchannels when compared to our previous study [36].

### Comparison to conventional experimental setups

**Fig. 5:**
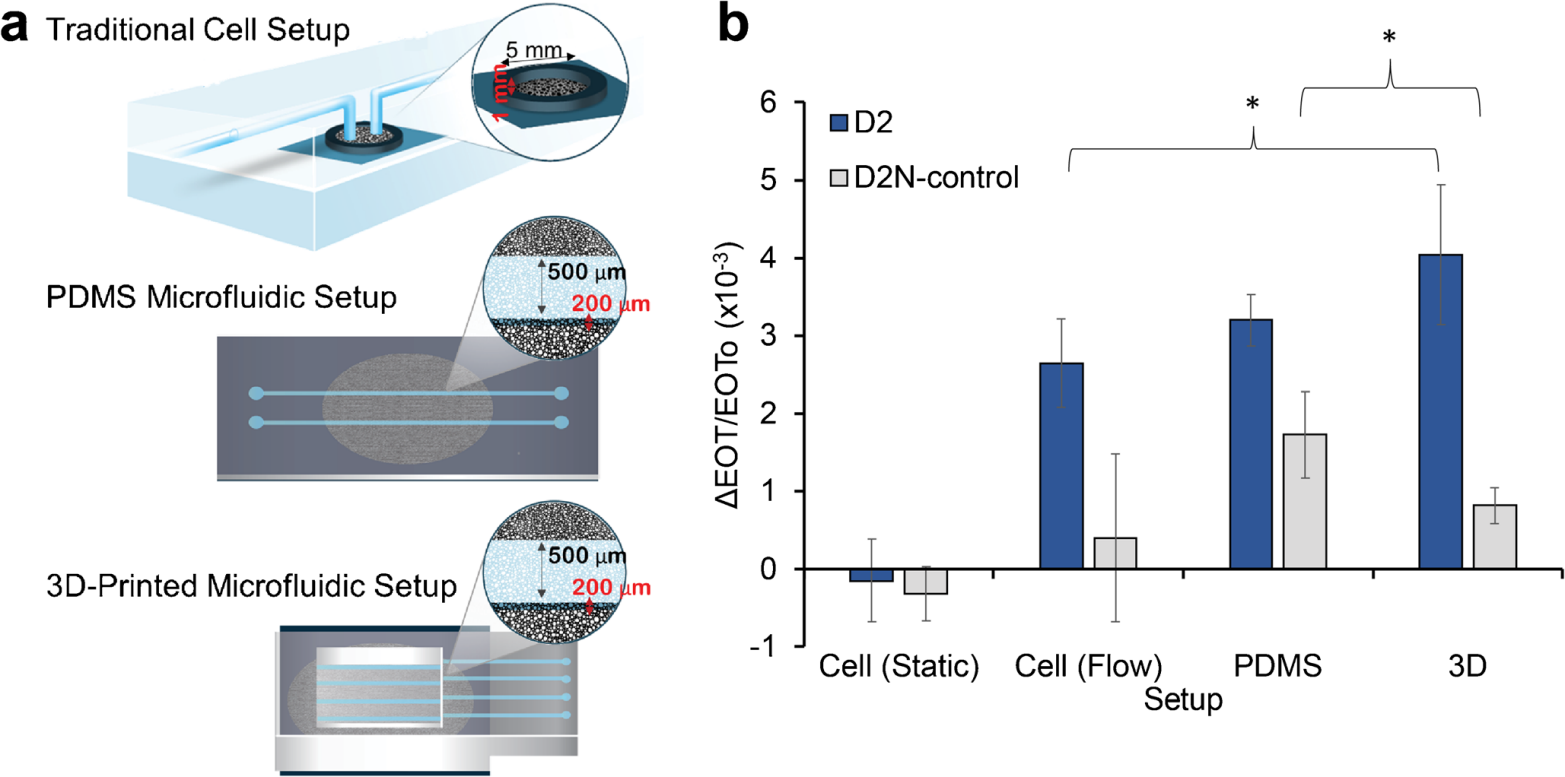
Comparison of the PSi aptasensor performance when integrated in different devices. **a** Schematics of the three experimental setups: 3D-printed microfluidics, PDMS microfluidics, and conventional cell (non-microfluidic); **b** Averaged relative EOT changes for detection of 1 μM D2 or D2N proteins in the three experimental setups. For the cell setup, biosensing experiments are performed in static (no flow) or flow configurations. The asterisk indicates a statistically significant difference (*t* test, *n* ≥ 3, *p* < 0.05)

The aptasensor performance in the 3D-printed microfluidic setup is compared to that observed when integrated in conventional experimental setups. The latter include a traditional cell setup [25, 36, 43] and PDMS microchannels with similar dimensions, as illustrated in Fig. 5a. The averaged relative EOT changes upon exposure to D2 and D2N proteins collected from the aptasensors, integrated in the different experimental setups, are presented in Fig. 5b. Results are presented for one protein concentration (1 μM), but are characteristic also of other concentrations. The 3D-printed microfluidic platform presents the highest signal for detection of the target D2 protein with a SNR ratio of 41 ± 17. Yet, its main advantage is in its selectivity, particularly when compared to the PDMS microfluidic setup; the latter presenting a 2.5-fold higher relative EOT signal for the non-target D2N control (*p* < 0.05). This suggests higher non-specific adsorption in the PDMS microfluidic setup, which also questions the aptamer functionality in this system. It should be noted that in both microfluidic systems, the aptamer immobilization step is performed in the microchannel, prior to the biosensing experiment. Table S2 (Supplementary information) compares point by point the construction process and performance of the 3D-printed and PDMS-based microfluidic aptasensors. For the PDMS microfluidic setup, aptamer immobilization in the microchannel significantly increases the EOT signal for both the target and non-target proteins, compared to aptamer immobilization prior to microchannel integration, see Figure S5 (Supplementary information). Moreover, the signal in the former case does not reach equilibrium within the time frame of the experiment. This behavior suggests different aptamer density within the PSi upon the two immobilization approaches and may be partly ascribed to the aptamer adsorption on the PDMS surface [44]. This in turn results in a lower aptamer density within the PSi upon aptamer immobilization in the PDMS microchannels, which exposes a larger surface area of the non-modified PSi nanostructure to non-specific adsorption of proteins [36]. Thus, although a similar signal is obtained for the target in the 3D-printed and PDMS microfluidic systems, in the PDMS channels, it is ascribed in part to non-specific protein adsorption on the PSi surface. The integration process of the PSi and the PDMS, including corona surface treatment and high temperature curing, may also affect the surface chemistry of the PSi. This emphasizes the advantage of the 3D-printed microfluidic platform and the developed bonding method, which avoids harsh conditions. It should be noted that the negative charge of the polyacrylate-based material used for the 3D printing may induce non-specific adsorption of positively charged biomolecules on its surface. Yet, as aptamers are negatively charged, they are not adsorbed to the 3D-printed microchannels and thus the aptasensor fabrication is not affected.

Our results also demonstrate the significant role of convection in these aptasensors, as target flow induces a higher signal in all systems, compared to a cell system without flow (see Fig. 5b and Table 1). The induced convection improves the mass transfer of the target to the aptasensor surface [3, 5], which can reach up to 25-fold higher target flux compared to a diffusion-based system, based on a theoretical calculation (see detailed calculation in the Supplementary information). This correlates with the ~70-fold enhancement in LOD compared to previous work [37], whereas further enhancement is ascribed to the smaller microchannel dimensions and the microchannel uneven edges of the 3D-printed platform (see Fig. 2c). The latter may contribute to solution mixing in the microchannel on top of the aptasensor, thus further improving the mass transfer [4].

## Conclusions

In this work, we present a simple and facile method for the integration of PSi-based aptasensors with 3D-printed polyacrylate microfluidics. The integration of both materials is based on a stamping technique with a UV-curable adhesive at room temperature. Successful bonding of the two substrates is demonstrated with a thin adhesive layer (~5 μm) in between; while the delicate porous regions within the microchannel remain clean and intact. As a proof-of-concept, we successfully immobilize a well-characterized anti-his tag aptamer as capture probe within the porous nanostructure, integrated in the microfluidic device, and demonstrate selective detection of a model target protein, compared to several non-target proteins, as well as complex *E. coli* lysate samples. The sensitivity of the integrated aptasensor, with a calculated LOD of 40 nM, is improved by ~70-fold compared to previous work. Thus, the developed bonding method does not impair the performance of the constructed biosensor. Furthermore, the resulting biosensor exhibits a superior selectivity and a higher detection signal for the target while integrated in the 3D-printed microfluidics, in comparison to the gold standard PDMS-based microfluidic setup with equal microchannel dimensions. For the latter, non-specific binding of the aptamer capture probe to the PDMS impairs the biosensors selectivity. Yet, the resolution of 3D printers, which dictates the microchannel dimensions, is currently on the range of tens of microns and much larger than applicable for PDMS-based microfluidics fabricated by soft lithography techniques. Moreover, the bonding method may also require adjustment to lower dimension microchannels or complex microstructures, due to the rough surface of the 3D-printed material, obtained due to the printer resolution. Nevertheless, 3D printing technology is rapidly advancing, the resolution keeps improving, and there are already printers with a resolution in the lower micrometer range.

The superior performance of the 3D-printed microfluidic integrated aptasensor in combination with its straightforward design and construction pave the way towards a more flexible approach to designing and investigating sophisticated microfluidic platforms integrated with PSi-based biosensors. For example, the presented microfluidic design could be adapted for multiplexed analyte detection, and integrated with different 3D-printed functional elements, such as pumps, valves, and mixing components, which facilitate device automation, portability, and high throughput [45]. These can be readily coupled with PSi aptasensors using the presented bonding method, where these aptasensors can be designed for detection of various target molecules simply by changing the aptamer capture probe, promoting the platform applicability in medical diagnostics [27, 46] and food quality and safety [47], as well as environmental monitoring.

## Supplementary information

ESM 1 (PDF 742 kb).
