# Supplementary material for: 3D-printed microfluidics integrated with optical nanostructured porous aptasensors for protein detection
Source: Mikrochim Acta. 2021 Feb 4;188(3):67. doi: 10.1007/s00604-021-04725-0 (PMC7862519; doi:10.1007/s00604-021-04725-0)
Supplement: Supplementary file 1 — (PDF 742 kb). [file 604_2021_4725_MOESM1_ESM.pdf]

Supplementary Information for

3D-Printed Microfluidics Integrated with Optical Nanostructured Porous Aptasensors for Protein Detection

Sofia Arshavsky-Graham [1,2], Anton Enders [2], Shanny Ackerman [1], Janina Bahnemann [2*], and Ester Segal [1,3*]

[1]Technion – Israel Institute of Technology, Department of Biotechnology and Food Engineering, Haifa, Israel

[2]Leibniz University Hannover, Institute of Technical Chemistry, Hanover, Germany

[3]Technion – Israel Institute of Technology, The Russell Berrie Nanotechnology Institute, Haifa, Israel

Additional Information Regarding Experimental Procedure

Protein Production and Purification

The target his tagged protein, domain 2 of extracellular endo-alpha-(1->5)-L-arabinanase 1 (from *Geobacillus stearothermophilus T-6*) (D2), and the non-target version without his tag (named D2N), were both produced and purified using the following method. *E. coli* BL21 cells, transformed with an abnA-D2 gene, were grown overnight at 37 °C on LB plates with appropriate antibiotic. The cells were transferred to a TB (Terrific Broth) medium for overnight growth in a shaker (230 rpm) at 37 °C. The cells were then cooled on ice for 15 min, harvested by centrifugation, and re-suspended in binding buffer (20 mM imidazole, 0.5 M NaCl, and 20 mM phosphate buffer, pH 7.4). Subsequently, the cells were disrupted by two passages through an Avestin Emulsiflex C3 Homogenizer, 1500 psi (Avestin, Ottawa, Canada) at room temperature, and centrifuged at 7000 rpm for 30 min. The supernatant was then heated at ~50 °C for 30 min and centrifuged again. The his-tagged protein was purified by fast protein liquid chromatography (FPLC) using the ÄKTA Avant-25 chromatography system (GE Healthcare Life Sciences) equipped with HisTrap column (1- or 5-ml column volume, GE Healthcare Life Sciences). The protein was eluted with a 10 CV gradient of elution buffer that contained 0.5 M imidazole, 0.5 M NaCl, and 20 mM phosphate buffer, pH 7.4. The protein D2N, without the his-tag, was purified via gel filtration using a Superdex 200 26/60 column, ÄKTA Avant (GE Healthcare Life Sciences), running at 2.5 ml/min with 50 mM Tris-HCl buffer pH 7.0, 100 mM NaCl and 0.02% sodium azide. Injected samples volumes were ~14 ml (~5% column volume).

Preparation of *E. coli* Lysates

E. coli K12 was cultivated in LB medium overnight under continuous shaking at 37 °C. The culture was spun down in a standard lab centrifuge (2-16P, Sigma Laboratory Centrifuges, Sigma-Aldrich) at 4500 rpm for 10 min, and the supernatant was replaced with SB buffer. This step was repeated twice. Subsequently, the culture was ultrasonicated on ice in a Vibra cell VCX 750 instrument (Sonics & Materials Inc., Newtown, Connecticut, USA) for 2 min, with 30 s pulses and 30 s pauses in between. The temperature and the amplitude were set to 4 °C and 40%, respectively. Cell debris were removed by ultracentrifugation at 4 °C for 30 min at 12000 g (1-15K, Sigma Laboratory Centrifuges, Sigma-Aldrich), and the supernatant was analyzed for protein content in NanoDrop instrument (NanoDrop 2000 spectrophotometer, Thermo Scientific), assuming 1 ABS=1 mg mL^{-1}. For the experiments, the lysate was diluted for protein content of 1 mg mL^{-1}.

Fabrication of Oxidized PSi Nanostructures

PSi Fabry-Pérot thin films were fabricated from a highly doped p-type crystalline Si wafers, using a two-step anodic electrochemical etching process, as previously described [1]. First, a sacrificial layer was etched at a constant current density of 375 mA cm^{-2} for 30 s in a 3:1 (v/v) solution of aqueous HF (48%) and ethanol, respectively. The resulting porous layer was removed by exposure to 0.1 M NaOH for 2 min, followed by a 1 min exposure to a solution of 1:3:1 (v/v) HF, ethanol and ddH_2O, respectively. Next, a second etching was conducted, at the same etching conditions as above. After each step, the silicon surface was thoroughly rinsed with ethanol and dried under nitrogen stream. The freshly etched PSi was thermally oxidized in a tube furnace (Thermo Scientific, Lindberg/Blue M™ 1200 °C Split-Hinge) at 800 °C for 1 h in ambient air, resulting in an oxidized PSi scaffold [2].

Post Processing of 3D-Printed Microfluidic Devices

Following printing, after the devices were cooled down, they were placed in a heat steam bath (EasyClean unit, 3D Systems, Rock Hill, USA) at 65 °C for 30 min and were subsequently immersed in hot biological oil bath (EasyClean unit, 3D Systems, Rock Hill, USA) at 65 °C for 30 min, to remove the support material. Next, hot oil was introduced into the channels using a syringe to remove any residues of support material. Finally, the devices were sonicated at 60 °C for 30 min in deionized water with detergent (Fairy Ultra Plus, Procter and Gamble, CT, USA) in an ultrasonic bath (Bandelin electronic, Berlin, Germany), followed by wash with 70% EtOH.

Experimental Setup

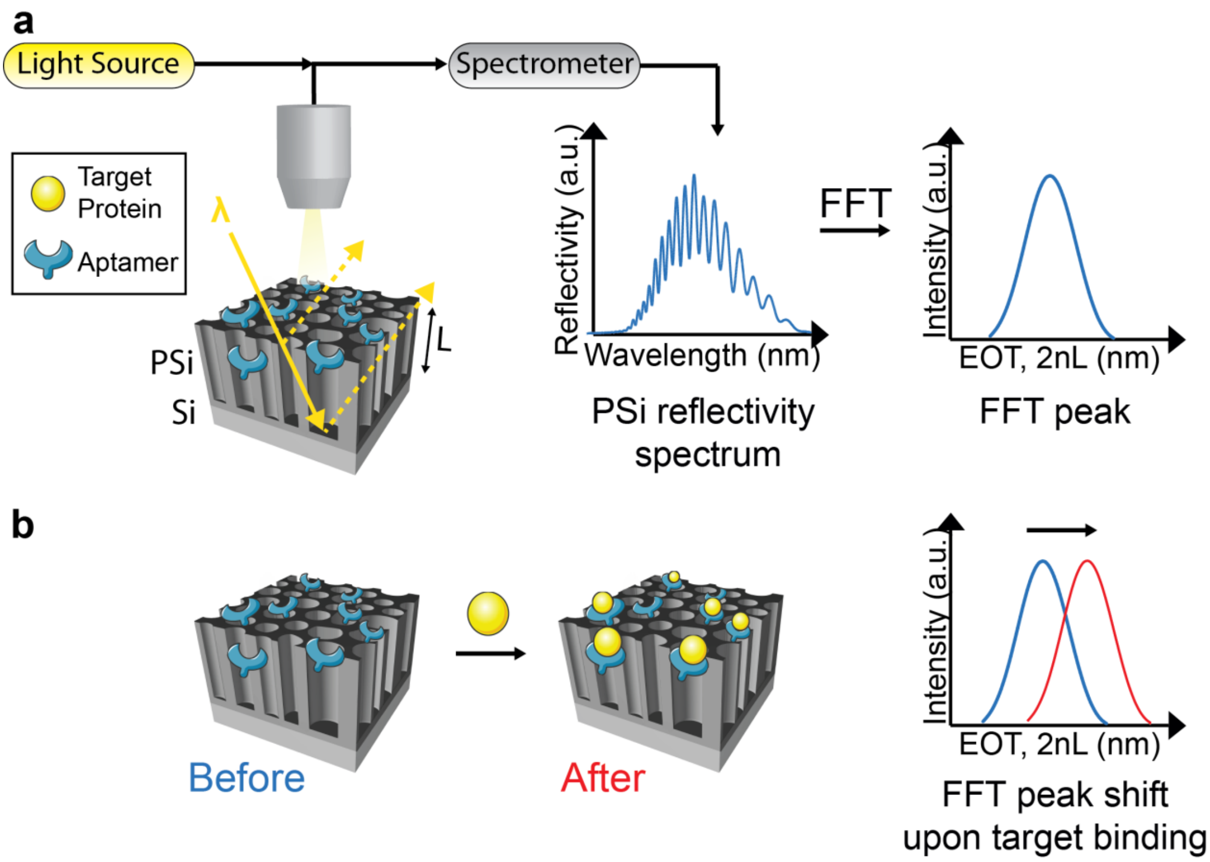

Figure S1. *A schematic of the optical signal acquisition and analysis. (a) The PSi film is illuminated with white light from a broadband light source and the reflectivity is monitored with a spectrometer, presenting a Fabry-Pérot fringe pattern due to interference from the two reflective interfaces of the PSi film. Application of Fast Fourier transformation (FFT) to the reflectivity spectrum results in a single peak, which position along the x axis equals to the effective optical signal (EOT) of the porous layer and linearly correlates to its average refractive index (n). (b) Upon target binding, an increase in the average refractive index of the PSi is obtained, observed as a shift of the FFT peak along the x-axis towards higher EOT values.*

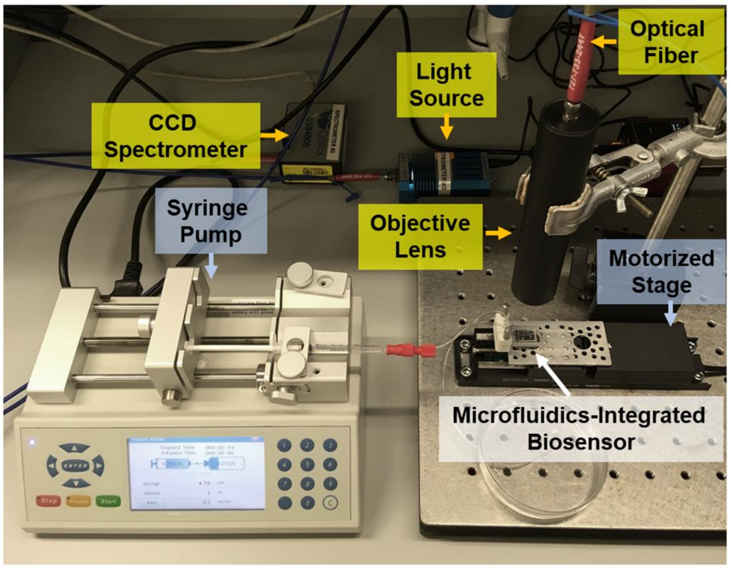

Figure S2*. Image of the 3D-printed microfluidic experimental setup.*

Porous Silicon Nanostructure Characterization

Porous layer thickness and porosity are evaluated by spectroscopic liquid infiltration method (SLIM). Briefly, the interferometric reflectance spectrum of the porous film is measured in air and while immersed in ethanol and acetone, having refractive indices of 1.359 and 1.357, respectively. The refractive index of the silicon oxide portion is assumed to be 1.455. The optical parameters from the reflectance spectra are then fitted to a Bruggeman effective medium approximation, yielding the thickness and the porosity of the porous layer.

Table S1. *Oxidized PSi nanostructure fabrication and characterization with SLIM (n=5)*

Wafer Resistivity (mΩ cm)	Current Density (mA cm^{-2})	Etching Time (s)	Porosity (%)	Porous Layer Thickness (μm)
0.90-1.00	375	30	73±3	5.5±0.2

Integrity of Bonded Devices

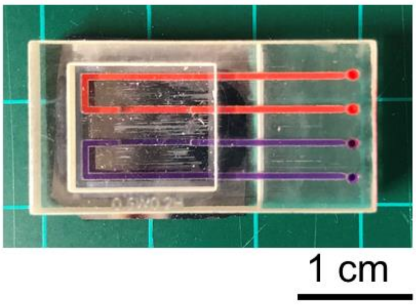

Figure S3. *The integrated 3D-printed microfluidic aptasensor with a dyed solution flowed within the microchannels >1-year post bonding, demonstrating the integrity of the device.*

Aptamer Immobilization

Aptamer immobilization can be conducted in two paths: either prior to microfluidic integration, or inside the microchannel following the integration. Fig. S4 presents a comparison of the optical signal of the two chemical immobilization paths, for the target D2 protein and the non-target D2N control (D2 without his-tag). When the aptamer immobilization is carried out inside the microchannel, a good signal is observed for the target protein compared with the non-target control. By contrast, when performing the immobilization prior to microfluidic integration, a similar biosensing signal is observed for both proteins. We ascribe this to a non-specific binding to the modified PSi surface, due to a poor aptamer functionality upon exposure to UV light during the microfluidic integration process.

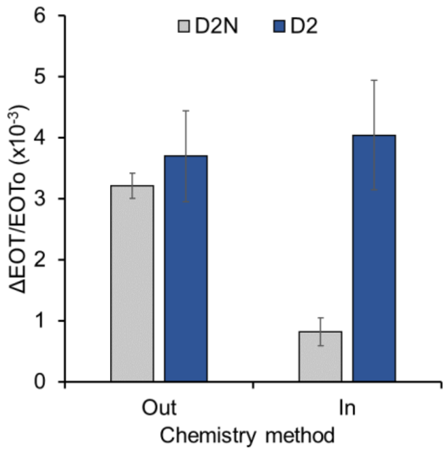

Figure S4. *Averaged relative EOT changes for the detection of 1 µM D2 (target) and D2N (non-target) proteins in the 3D-printed microfluidic integrated aptasensor, where the aptamer molecules are immobilized prior (chemistry out) or after (chemistry in) the microfluidic integration.*

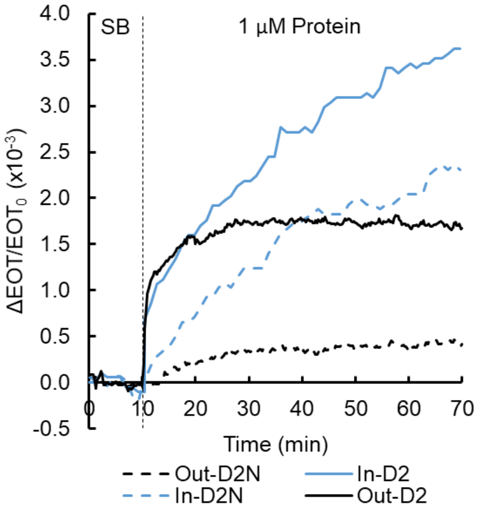

Figure S5. *Real time relative EOT changes upon introduction of D2 (target) or D2N (non-target) protein solutions (1 μM) to the PDMS microfluidic setup, in which the aptamer is immobilized prior to microfluidic integration (out) or within the microchannel (in).*

Derivation of Analyte Flux to the PSi Biosensor Surface in Static and Flow Experiments

The analyte flux to the PSi biosensor surface is calculated based on the thorough derivation of Squires *et al.* [3] and Lynn *et al.* [4]. In our calculation, we consider only the diffusion and convection in the bulk solution towards the surface. The microchannel system is characterized with a microchannel height (H) of $200 \cdot 10^{-6}$ m and a microchannel width (W) of $500 \cdot 10^{-6}$ m. The length from the inlet to the PSi biosensor (L) is 0.017 m. The target diffuses in the bulk solution with a diffusion coefficient (D) of $7 \cdot 10^{-11}$ m^2 s^{-1} [5]. The flow rate (Q) used for the experiments is $5 \cdot 10^{-10}$ m^3 s^{-1}. At steady state, an analyte concentration boundary layer with a thickness δ is formed above the biosensor surface. At a distance greater than δ, the analyte will have no interaction with the surface and will be swept away, while only molecules below δ will interact with the surface. While assuming the fluid flow can be approximated as a one-dimensional pressure-driven flow between two flat plates, the following relation for δ is obtained, as derived by Lynn *et al* [4]:

$$1) \quad \delta \approx L\left(\frac{DH^2W}{6L^2Q}\right)^{1/3}$$

and the flux of the analyte towards the biosensor surface is given by:

$$2) \quad J_{Diffusion+convection} \approx C_{A,0}\left(\frac{6D^2Q}{LH^2W}\right)^{1/3}$$

Where $C_{A,0}$ is the introduced analyte concentration. This expression gives an upper limit for the flux at the biosensor surface since it neglects the diffusion within the porous layer. For the flow experimental system, with introduced analyte concentration of $1 \cdot 10^{-6}$ M, the boundary layer thickness, δ, is estimated as $2 \cdot 10^{-5}$ m and the analyte $J_{Diffusion+convection}$ is estimated as $3.5 \cdot 10^{-9}$ mol m^{-2} s^{-1}.

For a system without convection, where the analyte solution is statically incubated on top of the biosensor, the flux can be simplified and described by [4, 3]:

$$3) \quad J_{Diffusion} \approx D(C_{A,0} - C_{A,s}) / \delta(t)$$

where $C_{A,s}$ is the analyte concentration at the sensor surface. δ in that case is not constant; it increases with the progress of analyte binding by the biosensor and scale as $\delta \sim \sqrt{Dt}$ [3]. We will assume that $C_{A,s}$ is zero for simplification, thus resulting in an upper limit for the flux, not considering the diffusion within the porous layer. Thus, the analyte flux at the biosensor surface in a static experiment will be:

$$4) \quad J_{Diffusion} \approx \frac{DC_{A,0}}{\sqrt{Dt}}$$

By comparing equations (2) and (4), the time when the fluxes will be equal in both systems can be derived, as:

$$5) \quad t \approx \frac{D}{\left(\frac{6D^2Q}{LH^2W}\right)^{2/3}}$$

which in our system equals to 5.7 s. For longer times, the flux in the static system will substantially decrease in comparison to the flow system. For instance, after 60 min of incubation with the analyte, which is the binding time used in our system, the bulk diffusion flux in the static system will be $1.4 \cdot 10^{-10}$ mol m^{-2} s^{-1}, more than an order of magnitude lower compared to the flow system.

Comparison of 3D-printed and PDMS-based microfluidic integrated aptasensors

***Table S2**. Overview of the construction of 3D-printed and PDMS-based microfluidic-integrated aptasensors and their biosensing performance.*

	**3D-Printed**	**PDMS**
Template	No template	3D-printed
Resolution	32 µm (lowest microchannel dimensions 64 µm)	Similar upon 3D-printed template.<br>Better for photolithographed template (lowest microchannel dimensions <10 µm)
Pre-bonding steps	Post printing processing steps, 2.5 h	PDMS curing at 60°C overnight
Bonding to PSi	Room temperature<br>UV curing for 30 min	Corona treatment<br>Baking at 90°C for 4 h
Aptamer immobilization	Within microchannels	Within microchannels
Average system noise ($\Delta EOT/EOT_0$ x10^3)	0.09	0.07
Optical signal for 1 µM target protein ($\Delta EOT/EOT_0$ x10^3)	4.0±0.9	3.2±0.3
SNR	41±17	53±12
%RSD	22	10
Optical signal for 1 µM non-target protein ($\Delta EOT/EOT_0$ x10^3)	0.8±0.2	1.7±0.6

References

1. Sailor MJ. Porous silicon in practice: preparation, characterization and applications. John Wiley & Sons; 2012.

2. Kilian KA, Bocking T, Gooding JJ. The importance of surface chemistry in mesoporous materials: lessons from porous silicon biosensors. Chemical Communications. 2009(6):630-40. doi:10.1039/B815449J.

3. Squires TM, Messinger RJ, Manalis SR. Making it stick: convection, reaction and diffusion in surface-based biosensors. Nature Biotechnology. 2008;26(4):417-26. doi:10.1038/nbt1388.

4. Lynn NS, Šípová H, Adam P, Homola J. Enhancement of affinity-based biosensors: effect of sensing chamber geometry on sensitivity. Lab on a Chip. 2013;13(7):1413-21. doi:10.1039/C2LC41184A.

5. Tyn MT, Gusek TW. Prediction of diffusion coefficients of proteins. Biotechnology and Bioengineering. 1990;35(4):327-38. doi:10.1002/bit.260350402.
